# Role of Erythrocytes in Nitric Oxide Metabolism and Paracrine Regulation of Endothelial Function

**DOI:** 10.3390/antiox11050943

**Published:** 2022-05-11

**Authors:** Damian Gajecki, Jakub Gawryś, Ewa Szahidewicz-Krupska, Adrian Doroszko

**Affiliations:** Clinical Department of Internal Medicine, Hypertension and Clinical Oncology, Wroclaw Medical University, Borowska 213, 50-556 Wroclaw, Poland; damian.gajecki@umw.edu.pl (D.G.); jakub.gawrys@umw.edu.pl (J.G.); ekrupska@gmail.com (E.S.-K.)

**Keywords:** red blood cells, nitric oxide, nitrates, nitrites, hemoglobin, endothelium, nitrosylation

## Abstract

Emerging studies provide new data shedding some light on the complex and pivotal role of red blood cells (RBCs) in nitric oxide (NO) metabolism and paracrine regulation of endothelial function. NO is involved in the regulation of vasodilatation, platelet aggregation, inflammation, hypoxic adaptation, and oxidative stress. Even though tremendous knowledge about NO metabolism has been collected, the exact RBCs’ status still requires evaluation. This paper summarizes the actual knowledge regarding the role of erythrocytes as a mobile depot of amino acids necessary for NO biotransformation. Moreover, the complex regulation of RBCs’ translocases is presented with a particular focus on cationic amino acid transporters (CATs) responsible for the NO substrates and derivatives transport. The main part demonstrates the intraerythrocytic metabolism of L-arginine with its regulation by reactive oxygen species and arginase activity. Additionally, the process of nitrite and nitrate turnover was demonstrated to be another stable source of NO, with its reduction by xanthine oxidoreductase or hemoglobin. Additional function of hemoglobin in NO synthesis and its subsequent stabilization in steady intermediates is also discussed. Furthermore, RBCs regulate the vascular tone by releasing ATP, inducing smooth muscle cell relaxation, and decreasing platelet aggregation. Erythrocytes and intraerythrocytic NO metabolism are also responsible for the maintenance of normotension. Hence, RBCs became a promising new therapeutic target in restoring NO homeostasis in cardiovascular disorders.

## 1. Human Erythrocytes as the Storage Pool of Amino Acids

Erythrocytes (red blood cells, RBCs) are the most numerous formed elements in human blood. Over the last several decades they have been considered to be responsible for gas exchange, as they transport oxygen and partly carbon dioxide. Even though RBCs are enucleated and lack numerous organelles, they consist of up to 2650 proteins, with at least 41 membrane transporters [1]. Hence, recently, a more complex function of RBCs has been postulated. It was demonstrated that RBCs play an important role in the transport of amino acids. In a vast majority of cases, amino acids’ concentrations in RBCs and plasma are relatively equal. Nevertheless, cationic amino acids, including L-citrulline, L-lysine, L-histidine and L-arginine (L-Arg), dominate in plasma, whereas L-ornithine—in the erythrocyte compartment [2].

Studies by Thorn et al. [3] confirmed that RBCs are capable of exchanging up to 15–17% of the total erythrocyte pool of amino acids with plasma, without subsequent alteration of the cellular osmotic balance. Moreover, in some in vitro studies, extended incubation of RBCs in plasma did not result in further intercompartmental exchange. It suggests that RBCs are the storage that could easily supply amino acids to deficient tissues as they circulate through capillaries and act as an inter-organ transporter.

## 2. RBC—The Importance of Transmembrane Translocases

So far, seven different amino acid transmembrane transport systems have been identified in erythrocytes. Four of them are based on facilitated diffusion (y+, y+L, L, T), and three constitute secondary active transporters (ASC, Gly, N) [3]. Interestingly, none of these systems have been found to transport threonine or methionine. The first characterized in RBCs was the y+L transport system, which binds cationic and neutral amino acids (leucine, lysine) and exhibits Na^+^-dependence. L transport is sodium-independent and transfers neutral amino acids, while the T transport system binds to tryptophan. Since not all of them are pivotal for regulating NO metabolism, in this review, only the main ones are discussed. As far as the literature is concerned, the most-studied systems in human erythrocytes are cationic amino acid transporters (CATs), presenting high structural homology to the classical amino acid transport system y+. CATs maintain the Na^+^- and pH-independent transport of cationic amino acids (CAAs), determined by transmembrane amino acids’ gradient. They are sensitive to trans-stimulation and saturable with amino acids plasma concentration. CATs are relatively selective for CAAs, including L-arginine, L-lysine, and L-ornithine. The family of CATs includes CAT1, CAT2a, CAT 2b, CAT3, and CAT4, where the first three have been demonstrated to transport CAAs through the erythrocyte membrane. CAT1 is characterized by the highest quantitative L-arginine transport [4]. CATs regulate the transmembrane CAAs arrangement, thereby controlling the intracellular metabolic processes. It has been proven that CATs undergo adaptive regulation based on the molecules’ availability. As the depletion of amino acids occurs, the protein membrane density increases in order to supply the CAAs more efficiently (adaptive de-repression). On the contrary, along with CAAs abundance, CATs express adaptive repression [5,6,7]. So far, this mechanism has been demonstrated in numerous human and animal cells. However, its exact role in erythrocyte transmembrane transport requires further confirmation.

Noteworthy, microRNAs (miRNAs) seem to be another CAT-1-regulating factor. The small noncoding RNAs consist of 21–25 nucleotides and act at the post-transcriptional level as negative regulators of mRNA expression. miRNAs bind to the target mRNA and cause translational repression or cleave mRNA sequences. Bhattacharyya et al. [8] reported that CAT-1 mRNA is regulated with miR-122. It is consistent with a recent study, which has demonstrated higher expression of miR-122 with subsequent decreased expression of CAT-1 among patients with hypertension and endothelial dysfunction [9]. Additionally, miR-122 level was proven to be positively correlated with markers of myocardial damage [10]. Furthermore, the use of miR-122 inhibitors seems to be a promising therapeutic target, as they reverse endothelial dysfunction [11]. Interestingly, miRNA-dependent CAT-1 downregulation is revised in case of amino acid starvation [8,12]. Contrary, the activity of CAT-1 is increased in cell stress, however, the pathomechanism is poorly understood [8,13,14].

Additionally, transmembrane polarization modulates CAT activity—hyperpolarization induces L-arginine cellular influx and increases erythrocyte L-arginine concentration.

## 3. Transport of the Selected Nitric Oxide Metabolic Pathway Intermediates between Erythrocytes and Plasma

The CATs-dependent concentration of L-Arginine regulates nitric oxide (NO) synthesis, as erythrocytes have been shown to express the two subtypes of nitric oxide synthase—endothelial and inducible (eNOS and iNOS, respectively) [15]. Furthermore, CATs also influence NO synthase by transporting L-arginine derivates, including asymmetric dimethylarginine (ADMA) and symmetric dimethylarginine (SDMA). ADMA is a competitive inhibitor of nitric oxide synthase, and simultaneously with its enantiomer, is formed from methylarginine-rich proteins, such as histones during their degradation. Strobel et al. [4] pointed out that CAT1 manages intracellular ADMA influx at physiological concentrations. Although both molecules compete for CATs at physiological concentration, L-arginine is characterized by greater affinity to the translocases. Hence, the influx of L-Arg to RBCs is accompanied by simultaneous inhibition of ADMA and SDMA influx [4].

The literature is inconsistent in terms of generation and storage of ADMA in RBCs. Davids et al. [16] demonstrated that RBCs transport ADMA, which had been previously incorporated from plasma. ADMA concentration stays in equilibrium between the intra- and extracellular compartments and is rapidly interchangeable. Studies with protease and the proteasome inhibitors have proven that RBCs are able to produce ADMA by enzymatic proteolysis of methylated proteins by the 20S proteasome [17]. The 20S proteasome generates peptides consisting of seven to nine amino acids in length, which might be subsequently cleaved by proteases. Mature RBCs are characterized by minimal turnover of proteins, and oxidative damage persistently leads to proteasome activation and increased ADMA generation. Even though hemoglobin is the major protein source, some studies indicate the low degradation rate of hemoglobin due to lower susceptibility to enzymatic degradation [16,18]. Nevertheless, other proteins were proven to be the targets for 20S proteasome and to become a source of intra-RBCs ADMA.

No consensus has been made regarding the ADMA degradation pathway. David et al. [16] postulate that ADMA is subsequently transferred out of RBCs, since no degradation of ADMA in RBCs has been observed. As a result, dimethylarginine dimethylaminohydrolase (DDAH) may not be present in RBCs. Contrary to that, Yokoro et al. [19] confirmed that DDAH-1 and protein-arginine ethyl transferase (PMRT) are expressed in red blood cells. Similarly, Kang et al. [20] demonstrated the expression of DDAH and its activity in RBCs. Therefore, RBCs seem to transport free ADMA taken mostly from plasma. Nevertheless, the lysis of RBCs during oxidative stress makes RBCs the potential ADMA generators.

CAT-dependent L-Arg transport is complex and is also regulated by hormones. Progesterone was found to inhibit transmembrane transfer via both phosphorylated protein kinase Cα (PKCα) and extracellular signal-regulated kinases (ERK1/2). On the contrary, estrogens were proven to increase L-Arg influx by modulating the constitutive ERK 1/2 signaling pathways and display protective properties [21].

Other studies reported that thyroid hormones cause upregulation of CATs, as they participate in cardiovascular abnormalities observed in the course of thyroid disorders. Thyroxine or triiodothyronine activate the membrane αvβ3 integrin receptor and transduce the signal through the stimulation of Phosphoinositide 3-kinase (PI3K), mitogen-activated protein kinase (MAPKs), and the intracellular calcium-dependent signaling pathways. Finally, it leads to increased mRNA expression of L-arginine transporters [22]. Even though RBCs contain abundant ERK1/2, the mentioned processes were presented in the endothelium, and further studies are needed to prove their presence and importance in erythrocytes.

## 4. Intraerythrocytic Metabolism of L-Arg and Its Regulation

Once L-arginine is moved to the erythrocyte compartment, it might be metabolized by arginase 1 or nitric oxide synthase (NOS), leading to NO synthesis. The exact mechanism regulating the proportion of entrance to one of these two competing metabolic pathways is unknown. However, it was shown that there is a negative correlation between arginase activity and NO synthesis. Arginase is supposed to be an important NOS regulator.

Emerging data reveal that peroxynitrite (ONOO^−^) might be a key player in modulating NO^−^ bioavailability, as it enhances arginase activity in red blood cells. Enhanced arginase activity causes inadequate substrate availability and leads to NOS uncoupling [23]. NOS loses its ability to convert L-arginine to L-citrulline. Nevertheless, NOS remains capable of transferring an electron from NADH and donating it to oxygen, leading to superoxide (O^2−^) production. NO can react with superoxide, forming peroxynitrite [24]. ONOO^−^ itself can also directly lead to NOS uncoupling by dissolving dimeric NOS conformation [25]. Excessive reactive oxygen species’ (ROS) formation decreases NO bioavailability in different ways. First of all, ROS decrease NO production by an increase in arginase activity, leading to a lack of L-Arg, while NOS uncoupling reduces the reaction rate. Secondly, ROS enhance NO degradation, by a reaction with ROS, resulting in peroxynitrite. In line with these studies, the role of ONOO^−^ in endothelial dysfunction in diabetes mellitus patients was investigated. It was demonstrated that incubation of RBCs obtained from diabetic subjects with peroxynitrite scavenger (FeTTPS) completely reverses RBCs-induced endothelial dysfunction [26,27]. Additionally, RBCs from healthy subjects were treated with peroxynitrite, leading to increased arginase activity.

Even though RBCs possess antioxidative and redox systems able to maintain intracellular homeostasis and to prevent oxidative damage, these mechanisms may be insufficient in patients with cardiovascular diseases and extensive ROS production [28]. Peroxynitrate and arginase activity may have great importance in myocardial ischemia-reperfusion injury, since arginase inhibition might exert a cardioprotective effect [29]. Moreover, some studies show that arginase plays an important role in pathogenesis of endothelial dysfunction in hypertension, atherosclerosis, obesity, and in the course of ageing [24,30].

The exact mechanisms underlying the regulation of arginase with ONOO^−^ in RBCs remain unclear. However, Rho-kinase-dependent increase of argininase expression by ONOO^−^ seems to be essential [31,32]. Alternative modifications of arginase like S-nitrosylation, glycosylation, or phosphorylation may play an important role, but this hypothesis requires further investigation [33]. Kim et al. [34] proved that alternative splicing may also have an impact on arginase activity. Addition of 24 nucleotides in gene exon 3 cause incorporation of eight amino acids (Val–Thr–Gln–Asn–Phe–Leu–Ile–Leu) in a hydrophobic-polar-hydrophobic sequence, leading to structural changes and reducing enzymatic activity up to 50% [32,34].

Interestingly, estrogens were found to increase NO production in RBCs by inducing eNOS phosphorylation via interaction with estrogen receptors (ERs) and activation of the PI-3 kinase/AKT pathway. The distribution and function of ERs (estrogen receptor ERα and ERβ) are different in premenopausal women and men. In premenopausal women, ERα are mostly localized on the RBCs membrane, which is related with increased PI-3 kinase/AKT-dependent eNOS phosphorylation. On the contrary, in men, ERα are mainly identified in the cytoplasm. It might be indicative of the role of red blood cells in NO-mediated, sex-dependent differences in cardiovascular risk [35,36].

## 5. RBCs as a Source of NO. Mechanisms Underlying Homeostasis of Its Bioavailability

**Figure 1:**
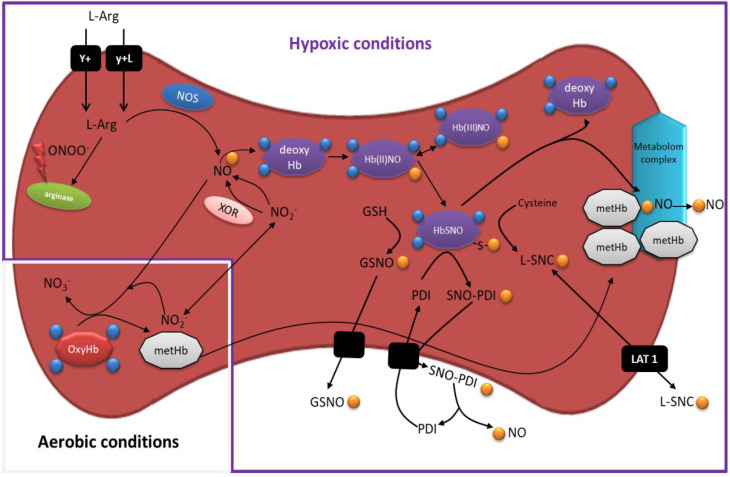
Nitric oxide metabolism in erythrocytes under aerobic and hypoxic conditions. In aerobic conditions, OxyHb reacts with NO, forming metHb and NO_3_^−^-metabolic end products. However, in a hypoxic environment, RBCs become a NO producer. L-arginine is transported through the RBCs membrane by y+ and yL. Subsequently, it is incorporated into NOS or the arginase pathway, depending on the redox RBCs status. NO reacts with deoxyHb and further undergoes S-nitrosylation, forming HbSNO. NO is finally transmitted to GSH, PDI, or cysteine and, as a steady intermediate, leaves the RBCs. Alternatively, NO_2_^−^ is reduced by XOR or deoxyHb and turned into another NO source. *Abbreviations: GSH: glutathione; GS-NO: S-nitrosoglutathione; HbSNO: S-nitroso hemoglobin; LAT 1: L-neutral amino acid transporter 1; L-SNC: S-nitroso-L-cysteine; SNO-PDI: S-nitrosylated protein disulphide isomerase; PDI: protein disulphide isomerase; XOR: xanthine oxidoreductase*.

As NO is a highly reactive molecule with an intravascular half-life of two milliseconds [37], for a long time it was considered to exert only an autocrine, local effect. NO reacts extremely fast (6–8 × 10^8^ M^−1^s^−1^) with oxyhemoglobin (oxyHb), forming methemoglobin (metHb) and nitrate (NO_3_^−^)-metabolic end products [38]. The oxidation of NO to NO_3_^−^ is protective against respiratory poisoning and nitrosative stress, although in pathological conditions, this mechanism reduces NO availability. For that reason, RBCs were initially considered to be NO scavengers, rather than a NO source. However, several studies revealed that RBCs are not only capable of producing NO but also able to store and transport NO to distal parts of the cardiovascular system (Figure 1).

Hemoglobin does not scavenge NO so easily, as it is surrounded with the cellular membrane, characterized by low permeability for endothelial NO [39]. Moreover, it was demonstrated that a pressure gradient created by the blood flow pushes RBCs to the center of the vascular lumen, forming a cell-free zone near the vascular wall [40]. In addition, it protects NO from scavenging by the cell-free hemoglobin released during hemolysis and makes downstream NO transport possible [39,41,42]. Furthermore, hemoglobin is a NO scavenger under normoxic conditions, but otherwise it manifests reductase activity [43].

Stamler et al. [44] revealed that NO is capable of binding to hemoglobin (Hb). However, this reaction depends on the Hb conformation and the magnitude of Hb oxygenation.

Hemoglobin exists in two allosteric conformations, which regulate Hb’s affinity to NO. The O_2_-dependent Hb quaternary conformation change is caused by allosteric anionic effectors, such as chloride, 2,3-diphosphoglycerate (DPG), and inositol hexaphosphate (IHP) [45]. While RBCs pass through pulmonary arteries, Hb binds two to three oxygen molecules and favors the R structure (relaxed oxyhemoglobin) [44]. On the contrary, in capillaries, it releases oxygen and changes the conformation to the T structure (tension-deoxyhemoglobin). Stamler demonstrated that R-state Hb binds NO and forms iron nitrosyl Hb (Hb (II)NO). The highest effectiveness of Hb reduction was observed with 50% Hb saturation. The reaction of binding NO to Hb is 5.6 times faster in the R-state, while NO releasing in the T-quaternary conformation increases 9.6-fold [46].

As NO is poorly bound to Hb, it is subsequently bound by S-nitrosylation of hemoglobin to the cysteine-93 residue, forming S-nitrosohemoglobin (HbSNO). This process is dependent on Hb conformation. The thiol affinity for NO is high in the R structure and low in the T structure [47]. This hypothesis was supported by demonstrating different HbSNO concentrations in venous and arterial RBCs [47].

As it results from other studies, nitrite seems to be another stable source of NO. Plasma is abundant with nitrite originating from dietary intake, endogenous synthesis, and inhalation of NO from the polluted air [48]. Once nitrite (NO_2_^−^) reaches the intra-RBCs compartment, it might be oxidized or reduced depending on the dominant oxygenation level and presumably the redox state. In well-oxygenated arterial blood, nitrite (NO_2_^−^) reacts with oxygen, forming nitrate (NO_3_^−^). It was shown that under hypoxic conditions, nitrites are reduced by deoxyhemoglobin, leading to NO regeneration. Subsequently, NO reacts with deoxy-Hb once more, as well as with other molecules, or is alternatively released to the plasma [49]. Concurrently, an alternative storage pool of NO becomes bioavailable, which might be easily mobilized when needed [50].

Contrary to the Stamler studies, Nagababu et al. [43] revealed that during the reduction of nitrates to nitrosohemoglobin, Hb(II)NO, the intermediate molecule is Hb(III)NO, which stays in equilibrium with Hb(II)NO. This discovery was of a great importance as it identified the pool of labile Hb(III)NO trapped in a hem pocket, which is not scavenged and can easily release NO under reductive conditions. To support this thesis, the authors demonstrated a relatively increased Hb(III)NO level in venous blood when compared to that of the arterial one. It may be considered as an indicator of increased nitrite reduction in capillaries, where hemoglobin becomes partially deoxygenated [43]. Keeping that in mind, nitrites (NO_2_^−^) might serve as a more important source of NO than S-nithrosothiols, especially in erythrocytes, being the major intravascular storage sites of nitrite in blood [51].

Other studies raise the different mechanism of nitrite conversion by xanthine oxidoreductase (XOR), located in the outer membrane of RBCs. In the study by Rathod et al. [52], the RBCs were incubated under acidic conditions with nitrate, xanthine, and a XOR or NOS inhibitor (allopurinol and L-N^G^-Nitro arginine methyl ester-L-NAME, respectively). It was demonstrated that NO generation was enhanced by ~43% in the presence of xanthine, pointing thus at the major role of XOR in NO synthesis in severe acidosis. Simultaneously, no difference was noted when the pH was in a normal range.

Another study with XOR inhibitor allopurinol demonstrated that although XOR activity is low under normal physiological conditions, it becomes important in pathological conditions, especially when pH drops to 6.8. In those conditions, XOR activity is responsible for ~2/3 of nitrite-derived NO production [53].

Dejam et al. [54] presented some other aspects of the role of XOR in regulating NO bioavailability. They showed concurrent nitrite reduction in normal physiology. The authors suggested that XOR inhibition increases NO bioavailability by reducing the ROS pool, produced by XOR in hypoxic conditions, finally leading to NO scavenging.

Other researchers provided some evidence that RBCs subjected to shear stress produce NO, which may have great importance while passing through vasoconstricted or stenotic vessels with a local response in NO secretion [55]. It was shown that RBCs are able to activate NOS after being exposed to shear stress [56]. Studies with eNOS inhibitors and extracellular EDTA calcium chelation show that intracellular calcium efflux through mechanosensitive RBC membrane channels provokes the binding of the calcium-calmodulin complex to the eNOS protein and its subsequent activation [57,58]. Further studies confirmed that this process is L-arginine-dependent and is highly limited in the presence of L-NAME [59].

The passage of erythrocytes through stenotic vessels and narrow capillaries in the microcirculation depends on RBCs deformability, which is also regulated by NO. One study, using eNOS inhibitors, demonstrated significant impairment of RBC deformability, which was reversed by exposure to exogenous NO donors [60]. The deformability is at least partially mediated by soluble guanylate cyclase (sGC) activation. Cortese-Krott et al. [61] revealed the existence of an sGC/PDE5/PKG-dependent signaling pathway, fully responsive to NO. The exact mechanism of regulation of the shape of RBCs by PKG is not well-studied, but K–Cl cotransport (COT) may be involved. It was proven that COT participates in the recovery of cell volume by modulating the cytoskeleton. As cell volume sensors detect deformation, they activate COT and induce regulatory volume decrease (RVD) [62]. NO is also involved in the regulation of eryptosis. This process may be triggered by different stimuli, leading to the opening of cation channels either directly or by protein kinase C with Ca^2+^. Erythrocyte shrinkage may be also induced by ceramide formation or by activation of some proteases [63]. NO displays a protective effect by stimulating cGMP-dependent protein kinase, resulting in a CA^2+^ influx decrease [64,65]. Additionally, NO is involved in the regulation of eryptosis via nitrosylation of eryptotic enzymes with NO to prevent RBCs lysis [65]. So far, the mechanism of NO migration from RBCs leading to induced smooth muscle cell relaxation, in order to restore blood flow and increase oxygen supply, remains unknown. Chen et al. [66] in an experimental study showed that the amount of NO transported from erythrocytes through free diffusion appears to be insufficient. It was demonstrated that in such an experimental setting, NO concentration would be below the half maximum effective concentration (EC50) for sGC and would not induce vasorelaxation. The question arises on how RBCs can supply enough NO to evoke the response of smooth muscle cells (SMCs). Another mathematic analysis discovered that the transport of inactive NO intermediates could be more effective and lead to a subsequent release of greater NO amounts. [66] Alternatively, facilitated NO transfer with membrane-bound proteins would also maintain RBC-dependent SMCs reaction [66].

Some authors suggest that Hb undergoes transnitrosylation again and that NO is transferred to glutathione (GSH), forming S-nitrosoglutathione (GSNO). GSNO may subsequently escape from RBCs via a transporter, release NO outside the erythrocytes, thus inducing vasorelaxation [50].

Opposite results were presented by Sandmann et al. [67], who claim that L-amino acids inhibit intracellular GSNO formation and postulate that it should not be considered as a vasoactive molecule. The authors suggest that S-nitrosocysteine might be a transfer molecule of NO out of RBC by the L-amino acids transporter system. Furthermore, Pawloski et al. [68] presented an alternative way of releasing NO from HbSNO. RBCs are postulated to form the cell lipid rafts that contain enzymes necessary in NO metabolism. The major component is the anion exchange protein 1 (AE1 or band 3 protein), which was proven to be an oxygen-sensitive molecular switch, that controls RBCs properties [69]. The quaternary conformation of Hb (R/T-structure) regulates the band 3 affinity to HbSNO. Along with decreased oxygen tension, N terminus of Band-3 associates reversibly with deoxyHb and the cytoplasmic domain of band 3 drives the transition from the R- to the T-state. It is accompanied with simultaneous NO transfer to the RBC membrane and facilitates the export of NO through membrane channels. The development of this hypothesis provided the study by Gladwin [70], according to which the erythrocytes contain the membrane rafts with complete “metabolome complex”(MCx) that facilitates NO effusion. MCx, besides the AE1/band 3, includes carbonic anhydrase, Rh, and aquaporin channels, and mixed hybrids of deoxyhemoglobin, methemoglobin, and carboxyhemoglobin that could catalytically amplify nitrite reduction. Additionally, local production of metHb in lipid rafts protects NO from scavenging by oxyHb [15,70]. Interestingly, emerging studies confirmed other interesting Band-3 properties. Its pivotal role was demonstrated in regulating RBCs’ glucose metabolism, cellular membrane stability, and ATP release [71]. In a hypoxic environment it promotes glycolysis over the pentose phosphate pathway, ATP-dependent vasodilatation, and increase RBCs’ deformability [69]. Band-3 was also found to transfer nitrate (NO_3_), nitrite (NO_2_), and peroxynitrite (OONO^−^) [72].

Alternatively, Dosier et al. [73] proved the significance of the L-neutral amino acid transporter 1 (LAT 1) in transferring S-nitroso-L-cysteine (SNC) out of the RBCs. LAT 1 is a high-affinity transport system for both cationic and neutral amino acids and exports cationic amino acids into the blood in exchange for large neutral amino acids [74]. It was confirmed that LAT 1 is expressed in the healthy RBC membrane, and LAT1 mRNA was identified in human reticulocytes. While using the LAT1-specific inhibitor (JPH-203), it was demonstrated that the blockage of LAT1 is connected with reduction in SNC efflux [73]. This mechanism could underlie the export of NO from RBCs, but its importance needs further investigation.

Another interesting theory was suggested by Kallakunta et al. [75] while analyzing the role of the protein disulphide isomerase (PDI) in NO transmembrane transport. PDI was detected in the membrane and in the cytosolic fraction of RBCs. The studies showed that RBCs acquire soluble PDI, which is released into the blood by various cells (hepatocytes, endothelial cells, leukocytes, platelet, pancreas). This protein is subsequently S-nityrosylated from SNO-Hb in cytosol and forms S-nitrosylated protein disulphide isomerase (SNO-PDI) [76]. Under normoxic conditions, SNO-PDI is transferred back through the membrane and stays attached to the external surface of RBCs. When RBCs reach a hypoxic environment, PDI is secreted in soluble form and interacts with the endothelium, triggering NO-dependent hypoxic vasodilation [75].

## 6. Erythrocyte-Dependent Paracrine Regulation of the Vascular NO Bioavailability

It is well-established that RBCs release adenosine triphosphate (ATP) and contain glycolytic enzymes required for its production [77,78]. It was proved that RBCs contain the gap junction protein pannexin 1 (Panx-1), which acts as an ATP-releasing channel in response to hypoxia, shear stress, or depolarization by forming transient channels between the intracellular compartment and the extracellular milieu [79,80]. Released ATP binds to P2Y receptors on erythrocytes and endothelium cells. P2Y receptors on RBCs mediate ATP-induced ATP release and lead to further increase in plasmatic ATP concentration [81]. Emerging data demonstrate that the P2Y12 inhibitor ticagrelor also induces ATP release from RBCs. The exact mechanism underlying ATP efflux from RBCs in response to that agent is not clear. However, it might be based on membrane channels (chloride channel or Panx-1), as ATP efflux decreases in the presence of some anion channel blockers [82]. The described pathway may explain the more potent anti-aggregative effect of ticagrelor in comparison to that of other antiplatelet agents, taking into consideration that increased extracellular level of adenosine (from ATP degradation) inhibits platelet aggregation additionally by A2A receptors [83].

Interestingly, when ATP reaches P2Y receptors and Panx-1 on endothelial cells, it evokes a calcium wave that propagates along the endothelium [84]. It leads to eNOS activation with associated a NO-dependent vasodilatory response, probably mediated by Ca^2+^-activated Cl^−^ channels and AMP-activated protein kinase (AMPK) [77,85,86,87]. Studies with Panx-1 inhibitors, such as carbenoxolone or probenecid, confirmed that ATP release is mostly mediated by Panx-1. Nevertheless, the inhibition was incomplete, suggesting the impact of other unknown mechanisms [88,89].

ATP released to the blood stream may also exert action by activating another subtype of purinergic receptors—P2X. In opposition to the P2Y receptors that protrude on the endothelium, P2X receptors are found on vascular smooth muscle cells (VSMCs). The direct stimulation of P2X with ATP may lead to local vasospasm [77].

Other transmembrane proteins participating in ATP transfer are the voltage dependent anion channels (VDACs). It was demonstrated that they are expressed on the RBCs’ membrane and create a complex with translocator protein 2 (TSPO2), the adenine nucleotide transporter (ANT) [90]. As activating ligands bind to the complex, they provoke calcium infusion and enhance cAMP-dependent signaling. Ca^2+^ and cAMP activate protein kinases C and A, respectively, which results in VDACs phosphorylation and polymerization, leading finally to ATP release through the complex [91]. Noteworthy, Sridharan et al. [89] pointed out that VDACs are also responsible for ATP release in response to prostacyclin analogues or β-adrenergic stimulation.

Numerous papers demonstrate that RBCs adjust the blood flow by a decrease in platelet aggregation. Srihirun et al. [92] reported that RBCs are mandatory for inhibiting ADP or collagen-induced platelet aggregation by releasing NO from nitrite. Nitrite alone has no effect on platelets, while reduction in platelet aggregation was observed in the presence of RBCs. The result was limited by adding a NO scavenger (C-PTIO) which confirmed the NO-dependent characteristics of this reaction. Additionally, the antiplatelet outcome was enhanced under hypoxic conditions, demonstrating thus the presence of reductase activity of deoxygenated hemoglobin.

Furthermore, it is well-documented that endothelial eNOS, via NO synthesis, affects systemic blood pressure (BP). Leo et al. [93] shed some light on the role of RBCs in preserving normotension in an eNOS-knockout mice model.

One of the reasons leading to a higher BP profile might be CAT-1 alteration, which is a risk factor for the development of hypertension. Yang et al. [94] studied the polymorphism in the 3′UTR region of CAT-1 chromosome 13q12-q14, which leads to altered L-arginine metabolism with hypertensive presentation. CAT-1 was also proven to be at least partially responsible for cardiovascular toxicity, with increased BP induced by cyclosporine. It was presented that cyclosporine significantly attenuates L-arginine transport and induces protein nitration, leading to the development of hypertension and accelerated atherosclerosis [95].

NO, released by RBCs to the blood stream, also becomes an autocrine agent that increases RBCs’ deformability. It is essential when RBCs pass through narrow capillaries in microcirculation. The exact way of this regulation is uncertain, however, NO may act by regulating guanylate cyclase, ion transport across the RBC membrane, or S-nitrosylation of cytoskeleton spectrins [96,97]. Oxidative stress also reduces RBCs deformability, which aggravates blood flow in the microvascular bed. In line with that, the abnormalities of hemorheological properties of RBCs were demonstrated to play a pivotal role in pathogenesis of microvascular angina (MVA). Furthermore, patients with MVA demonstrate an increased oxidated-to-reduced glutathione ratio (the GSSG/GSH index), suggesting a central role of oxidative stress in MVA development [98].

## 7. Future Perspectives

Over the past few years, significant progress regarding the knowledge on the role of erythrocytes in nitric oxide metabolism has been made. From simple gas transporters, through nitric oxide scavengers, RBCs have become important players in nitric oxide synthesis and in the maintenance of appropriate vascular tone. Erythrocytes seem to be a promising novel therapeutic target in management of endothelial dysfunction. Development of specific arginase inhibitors (AI) may bring benefits in patients with coronary artery disease, diabetes mellitus, heart failure, hypertension, and erectile dysfunction [99,100,101,102]. Studies on animal models demonstrated that increased activity of arginase has fundamental importance in myocardial ischemia-reperfusion injury and that its inhibition mediates cardiac protection via increased NO production [103,104,105]. Despite many in vitro studies, further clinical trials should be conducted to provide some more evidence on the usefulness of AI in cardiovascular risk reduction. Up to date, a promising trial with an arginase inhibitor (Nω-hydroxy-nor-L-arginine) was conducted, with restoration of endothelial function in patients with type 2 diabetes [106]. Similar results were obtained in endothelial dysfunction caused by ischemia-reperfusion in patients with CAD [107]. e-NOS uncoupling and prevention of oxidative stress seem to be other interesting future therapeutic targets.

## Data Availability

Not applicable.

## References

[1] Bryk A.H., Wiśniewski J.R.W. (2017). Quantitative Analysis of Human Red Blood Cell Proteome. J. Proteome Res..

[2] Agli A.N., Schaefer A., Geny B., Piquard F., Haberey P. (1998). Erythrocytes Participate Significantly in Blood Transport of Amino Acids during the Post Absorptive State in Normal Humans. Eur. J. Appl. Physiol. Occup. Physiol..

[3] Thorn B., Dunstan R.H., Macdonald M.M., Borges N., Roberts T.K. (2020). Evidence That Human and Equine Erythrocytes Could Have Significant Roles in the Transport and Delivery of Amino Acids to Organs and Tissues. Amino Acids.

[4] Strobel J., Mieth M., Endreß B., Auge D., König J., Fromm M.F., Maas R. (2012). Interaction of the Cardiovascular Risk Marker Asymmetric Dimethylarginine (ADMA) with the Human Cationic Amino Acid Transporter 1 (CAT1). J. Mol. Cell. Cardiol..

[5] Franchi Gazzola R., Sala R., Bussolati O., Visigalli R., Dall’Asta V., Ganapathy V., Gazzola G.C. (2001). The Adaptive Regulation of Amino Acid Transport System A Is Associated to Changes in ATA2 Expression. FEBS Lett..

[6] Gräf P., Förstermann U., Closs E.I. (2001). The Transport Activity of the Human Cationic Amino Acid Transporter HCAT-1 Is Downregulated by Activation of Protein Kinase C. Br. J. Pharmacol..

[7] Fernandez J., Bode B., Koromilas A., Alan Diehl J., Krukovets I., Snider M.D., Hatzoglou M. (2002). Translation Mediated by the Internal Ribosome Entry Site of the Cat-1 MRNA Is Regulated by Glucose Availability in a PERK Kinase-Dependent Manner. J. Biol. Chem..

[8] Bhattacharyya S.N., Habermacher R., Martine U., Closs E.I., Filipowicz W. (2006). Relief of MicroRNA-Mediated Translational Repression in Human Cells Subjected to Stress. Cell.

[9] Zhang H.-G., Zhang Q.-J., Li B.-W., Li L.-H., Song X.-H., Xiong C.-M., Zou Y.-B., Liu B.-Y., Han J.-Q., Xiu R.-J. (2020). The Circulating Level of MiR-122 Is a Potential Risk Factor for Endothelial Dysfunction in Young Patients with Essential Hypertension. Hypertens. Res..

[10] Wang L., Chen H. (2021). Correlation between Serum MiR-122 and Myocardial Damage and Ventricular Function in Patients with Essential Hypertension. J. Thorac. Dis..

[11] Gaddam R.R., Dhuri K., Kim Y.-R., Jacobs J.S., Kumar V., Li Q., Irani K., Bahal R., Vikram A. (2022). γ Peptide Nucleic Acid-Based MiR-122 Inhibition Rescues Vascular Endothelial Dysfunction in Mice Fed a High-Fat Diet. J. Med. Chem..

[12] Hatzoglou M., Fernandez J., Yaman I., Closs E. (2004). Regulation of Cationic Amino Acid Transport: The Story of the CAT-1 Transporter. Annu. Rev. Nutr..

[13] Doss J.F., Corcoran D.L., Jima D.D., Telen M.J., Dave S.S., Chi J.T. (2015). A Comprehensive Joint Analysis of the Long and Short RNA Transcriptomes of Human Erythrocytes. BMC Genom..

[14] Kabanova S., Kleinbongard P., Volkmer J., Andrée B., Kelm M., Jax T.W. (2009). Gene Expression Analysis of Human Red Blood Cells. Int. J. Med. Sci..

[15] Cortese-Krott M.M., Kelm M. (2014). Endothelial Nitric Oxide Synthase in Red Blood Cells: Key to a New Erythrocrine Function?. Redox Biol..

[16] Davids M., van Hell A.J., Visser M., Nijveldt R.J., van Leeuwen P.A.M., Teerlink T. (2012). Role of the Human Erythrocyte in Generation and Storage of Asymmetric Dimethylarginine. Am. J. Physiol.-Heart Circ. Physiol..

[17] Neelam S., Kakhniashvili D.G., Wilkens S., Levene S.D., Goodman S.R. (2011). Functional 20S Proteasomes in Mature Human Red Blood Cells. Exp. Biol. Med..

[18] Sitar M.E., Kayacelebi A.A., Beckmann B., Kielstein J.T., Tsikas D. (2015). Asymmetric Dimethylarginine (ADMA) in Human Blood: Effects of Extended Haemodialysis in the Critically Ill Patient with Acute Kidney Injury, Protein Binding to Human Serum Albumin and Proteolysis by Thermolysin. Amino Acids.

[19] Yokoro M., Suzuki M., Murota K., Otsuka C., Yamashita H., Takahashi Y., Tsuji H., Kimoto M. (2012). Asymmetric Dimethylarginine, an Endogenous NOS Inhibitor, Is Actively Metabolized in Rat Erythrocytes. Biosci. Biotechnol. Biochem..

[20] Kang E.S., Cates T.B., Harper D.N., Chiang T.M., Myers L.K., Acchiardo S.R., Kimoto M. (2001). An Enzyme Hydrolyzing Methylated Inhibitors of Nitric Oxide Synthase Is Present in Circulating Human Red Blood Cells. Free Radic. Res..

[21] Bentur O.S., Schwartz D., Chernichovski T., Ingbir M., Weinstein T., Chernin G., Schwartz I.F. (2015). Estradiol Augments While Progesterone Inhibits Arginine Transport in Human Endothelial Cells through Modulation of Cationic Amino Acid Transporter-1. Am. J. Physiol. Integr. Comp. Physiol..

[22] Toral M., Jimenez R., Montoro-Molina S., Romero M., Wangensteen R., Duarte J., Vargas F. (2018). Thyroid Hormones Stimulate L-Arginine Transport in Human Endothelial Cells. J. Endocrinol..

[23] Kietadisorn R., Juni R.P., Moens A.L. (2012). Tackling Endothelial Dysfunction by Modulating NOS Uncoupling: New Insights into Its Pathogenesis and Therapeutic Possibilities. Am. J. Physiol. Metab..

[24] Roe N.D., Ren J. (2012). Nitric Oxide Synthase Uncoupling: A Therapeutic Target in Cardiovascular Diseases. Vascul. Pharmacol..

[25] Zou M.-H., Shi C., Cohen R.A. (2002). Oxidation of the Zinc-Thiolate Complex and Uncoupling of Endothelial Nitric Oxide Synthase by Peroxynitrite. J. Clin. Investig..

[26] Mahdi A., Tengbom J., Alvarsson M., Wernly B., Zhou Z., Pernow J. (2020). Red Blood Cell Peroxynitrite Causes Endothelial Dysfunction in Type 2 Diabetes Mellitus via Arginase. Cells.

[27] Zhou Z., Mahdi A., Tratsiakovich Y., Zahorán S., Kövamees O., Nordin F., Uribe Gonzalez A.E., Alvarsson M., Östenson C.G., Andersson D.C. (2018). Erythrocytes from Patients with Type 2 Diabetes Induce Endothelial Dysfunction Via Arginase I. J. Am. Coll. Cardiol..

[28] Pernow J., Mahdi A., Yang J., Zhou Z. (2019). Red Blood Cell Dysfunction: A New Player in Cardiovascular Disease. Cardiovasc. Res..

[29] Yang J., Gonon A.T., Sjoquist P.-O., Lundberg J.O., Pernow J. (2013). Arginase Regulates Red Blood Cell Nitric Oxide Synthase and Export of Cardioprotective Nitric Oxide Bioactivity. Proc. Natl. Acad. Sci. USA.

[30] Mahdi A., Kövamees O., Pernow J. (2020). Improvement in Endothelial Function in Cardiovascular Disease-Is Arginase the Target?. Int. J. Cardiol..

[31] Romero M.J., Platt D.H., Tawfik H.E., Labazi M., El-Remessy A.B., Bartoli M., Caldwell R.B., Caldwell R.W. (2008). Diabetes-Induced Coronary Vascular Dysfunction Involves Increased Arginase Activity. Circ. Res..

[32] El-Remessy A.B., Tawfik H.E., Matragoon S., Pillai B., Caldwell R.B., Caldwell R.W. (2010). Peroxynitrite Mediates Diabetes-Induced Endothelial Dysfunction: Possible Role of Rho Kinase Activation. Exp. Diabetes Res..

[33] Santhanam L., Lim H.K., Lim H.K., Miriel V., Brown T., Patel M., Balanson S., Ryoo S., Anderson M., Irani K. (2007). Inducible NO Synthase–Dependent S -Nitrosylation and Activation of Arginase1 Contribute to Age-Related Endothelial Dysfunction. Circ. Res..

[34] Kim P.S., Iyer R.K., Lu K.V., Yu H., Karimi A., Kern R.M., Tai D.K., Cederbaum S.D., Grody W.W. (2002). Expression of the Liver Form of Arginase in Erythrocytes. Mol. Genet. Metab..

[35] Duckles S.P., Miller V.M. (2010). Hormonal Modulation of Endothelial NO Production. Pflügers Arch.-Eur. J. Physiol..

[36] Vona R., Gambardella L., Ortona E., Santulli M., Malorni W., Carè A., Pietraforte D., Straface E. (2019). Functional Estrogen Receptors of Red Blood Cells. Do They Influence Intracellular Signaling?. Cell. Physiol. Biochem..

[37] Liu X., Miller M.J.S., Joshi M.S., Sadowska-Krowicka H., Clark D.A., Lancaster J.R. (1998). Diffusion-Limited Reaction of Free Nitric Oxide with Erythrocytes. J. Biol. Chem..

[38] Helms C.C., Gladwin M.T., Kim-Shapiro D.B. (2018). Erythrocytes and Vascular Function: Oxygen and Nitric Oxide. Front. Physiol..

[39] Vaughn M.W., Huang K.-T., Kuo L., Liao J.C. (2000). Erythrocytes Possess an Intrinsic Barrier to Nitric Oxide Consumption. J. Biol. Chem..

[40] Chebbi R. (2019). A Two-Zone Shear-Induced Red Blood Cell Migration Model for Blood Flow in Microvessels. Front. Phys..

[41] Deonikar P., Kavdia M. (2010). A Computational Model for Nitric Oxide, Nitrite and Nitrate Biotransport in the Microcirculation: Effect of Reduced Nitric Oxide Consumption by Red Blood Cells and Blood Velocity. Microvasc. Res..

[42] Liao J.C., Hein T.W., Vaughn M.W., Huang K.-T., Kuo L. (1999). Intravascular Flow Decreases Erythrocyte Consumption of Nitric Oxide. Proc. Natl. Acad. Sci. USA.

[43] Nagababu E., Ramasamy S., Abernethy D.R., Rifkind J.M. (2003). Active Nitric Oxide Produced in the Red Cell under Hypoxic Conditions by Deoxyhemoglobin-Mediated Nitrite Reduction. J. Biol. Chem..

[44] Stamler J.S., Jia L., Eu J.P., McMahon T.J., Demchenko I.T., Bonaventura J., Gernert K., Piantadosi C.A. (1997). Blood Flow Regulation by S -Nitrosohemoglobin in the Physiological Oxygen Gradient. Science.

[45] Fago A., Crumbliss A.L., Hendrich M.P., Pearce L.L., Peterson J., Henkens R., Bonaventura C. (2013). Oxygen Binding to Partially Nitrosylated Hemoglobin. Biochim. Biophys. Acta.-Proteins Proteom..

[46] Rifkind J.M., Nagababu E., Ramasamy S. (2011). The Quaternary Hemoglobin Conformation Regulates the Formation of the Nitrite-Induced Bioactive Intermediate and the Dissociation of Nitric Oxide from This Intermediate. Nitric Oxide.

[47] Jia L., Bonaventura C., Bonaventura J., Stamler J.S. (1997). S-Nitrosohaemoglobin: A Dynamic Activity of Blood Involved in Vascular Control. Transfus. Med. Rev..

[48] Baylis C., Vallance P. (1998). Measurement of Nitrite and Nitrate Levels in Plasma and Urine—What Does This Measure Tell Us about the Activity of the Endogenous Nitric Oxite System?. Curr. Opin. Nephrol. Hypertens..

[49] Cosby K., Partovi K.S., Crawford J.H., Patel R.P., Reiter C.D., Martyr S., Yang B.K., Waclawiw M.A., Zalos G., Xu X. (2003). Nitrite Reduction to Nitric Oxide by Deoxyhemoglobin Vasodilates the Human Circulation. Nat. Med..

[50] Schmidt H.H.H.W., Feelisch M. (2019). Red Blood Cell–Derived Nitric Oxide Bioactivity and Hypoxic Vasodilation. Circulation.

[51] Dejam A. (2005). Erythrocytes Are the Major Intravascular Storage Sites of Nitrite in Human Blood. Blood.

[52] Rathod K.S., Webb A.J., Lovell M.J., Lecomte F., Ahluwalia A. (2006). Nitrite Is Reduced to Nitric Oxide by Xanthine Oxidoreductase and Nitric Oxide Synthase in the Erythrocyte Membrane in Hypoxemia. Blood.

[53] Webb A.J., Milsom A.B., Rathod K.S., Chu W.L., Qureshi S., Lovell M.J., Lecomte F.M.J., Perrett D., Raimondo C., Khoshbin E. (2008). Mechanisms Underlying Erythrocyte and Endothelial Nitrite Reduction to Nitric Oxide in Hypoxia. Circ. Res..

[54] Dejam A., Hunter C.J., Tremonti C., Pluta R.M., Yuen Y.H., Hon Y., Grimes G., Partovi K., Pelletier M.M., Oldfield E.H. (2007). Nitrite Infusion in Humans and Nonhuman Primates Endocrine Effects, Pharmacokinetics and Tolerance Formation. Circulation.

[55] Ulker P., Yaras N., Yalcin O., Celik-Ozenci C., Johnson P.C., Meiselman H.J., Baskurt O.K. (2011). Shear Stress Activation of Nitric Oxide Synthase and Increased Nitric Oxide Levels in Human Red Blood Cells. Nitric Oxide.

[56] Ulker P., Sati L., Celik-Ozenci C., Meiselman H.J., Baskurt O.K. (2009). Mechanical Stimulation of Nitric Oxide Synthesizing Mechanisms in Erythrocytes. Biorheology.

[57] Feron O., Saldana F., Michel J.B., Michel T. (1998). The Endothelial Nitric-Oxide Synthase-Caveolin Regulatory Cycle. J. Biol. Chem..

[58] Ulker P., Meiselman H.J., Baskurt O.K. (2010). Nitric Oxide Generation in Red Blood Cells Induced by Mechanical Stress. Clin. Hemorheol. Microcirc..

[59] Fleming I., Busse R. (2003). Molecular Mechanisms Involved in the Regulation of the Endothelial Nitric Oxide Synthase. Am. J. Physiol.-Regul. Integr. Comp. Physiol..

[60] Bor-Kucukatay M., Wenby R.B., Meiselman H.J., Baskurt O.K. (2003). Effects of Nitric Oxide on Red Blood Cell Deformability. Am. J. Physiol.-Heart Circ. Physiol..

[61] Cortese-Krott M.M., Mergia E., Kramer C.M., Lückstädt W., Yang J., Wolff G., Panknin C., Bracht T., Sitek B., Pernow J. (2018). Identification of a Soluble Guanylate Cyclase in RBCs: Preserved Activity in Patients with Coronary Artery Disease. Redox Biol..

[62] Adragna N.C., Fulvio M.D., Lauf P.K. (2004). Regulation of K-Cl Cotransport: From Function to Genes. J. Membr. Biol..

[63] Dreischer P., Duszenko M., Stein J., Wieder T. (2022). Eryptosis: Programmed Death of Nucleus-Free, Iron-Filled Blood Cells. Cells.

[64] Brun J.-F., Varlet-Marie E., Myzia J., de Mauverger E.R., Pretorius E. (2021). Metabolic Influences Modulating Erythrocyte Deformability and Eryptosis. Metabolites.

[65] Föller M., Feil S., Ghoreschi K., Koka S., Gerling A., Thunemann M., Hofmann F., Schuler B., Vogel J., Pichler B. (2008). Anemia and Splenomegaly in CGKI-Deficient Mice. Proc. Natl. Acad. Sci. USA.

[66] Chen K., Piknova B., Pittman R.N., Schechter A.N., Popel A.S. (2008). Nitric Oxide from Nitrite Reduction by Hemoglobin in the Plasma and Erythrocytes. Nitric Oxide.

[67] Sandmann J., Schwedhelm K.S., Tsikas D. (2005). Specific Transport of S -Nitrosocysteine in Human Red Blood Cells: Implications for Formation of S -Nitrosothiols and Transport of NO Bioactivity within the Vasculature. FEBS Lett..

[68] Pawloski J.R., Hess D.T., Stamler J.S. (2001). Export by Red Blood Cells of Nitric Oxide Bioactivity. Nature.

[69] Chu H., McKenna M.M., Krump N.A., Zheng S., Mendelsohn L., Thein S.L., Garrett L.J., Bodine D.M., Low P.S. (2016). Reversible Binding of Hemoglobin to Band 3 Constitutes the Molecular Switch That Mediates O_2_ Regulation of Erythrocyte Properties. Blood.

[70] Gladwin M.T., Schechter A.N., Kim-Shapiro D.B., Patel R.P., Hogg N., Shiva S., Cannon R.O., Kelm M., Wink D.A., Espey M.G. (2005). The Emerging Biology of the Nitrite Anion. Nat. Chem. Biol..

[71] Zhao Y., Wang X., Wang R., Chen D., Noviana M., Zhu H. (2019). Nitric Oxide Inhibits Hypoxia-induced Impairment of Human RBC Deformability through Reducing the Cross-linking of Membrane Protein Band 3. J. Cell. Biochem..

[72] Jennings M.L. (2021). Cell Physiology and Molecular Mechanism of Anion Transport by Erythrocyte Band 3/AE1. Am. J. Physiol. Physiol..

[73] Dosier L.B.M., Premkumar V.J., Zhu H., Akosman I., Wempe M.F., McMahon T.J. (2017). Antagonists of the System L Neutral Amino Acid Transporter (LAT) Promote Endothelial Adhesivity of Human Red Blood Cells. Thromb. Haemost..

[74] Verrey F., Closs E.I., Wagner C.A., Palacin M., Endou H., Kanai Y. (2004). CATs and HATs: The SLC7 Family of Amino Acid Transporters. Pflügers Arch.-Eur. J. Physiol..

[75] Kallakunta V.M., Slama-Schwok A., Mutus B. (2013). Protein Disulfide Isomerase May Facilitate the Efflux of Nitrite Derived S-Nitrosothiols from Red Blood Cells. Redox Biol..

[76] Ramachandran N., Root P., Jiang X.-M., Hogg P.J., Mutus B. (2001). Mechanism of Transfer of NO from Extracellular S-Nitrosothiols into the Cytosol by Cell-Surface Protein Disulfide Isomerase. Proc. Natl. Acad. Sci. USA.

[77] Bergfeld G.R., Forrester T. (1992). Release of ATP from Human Erythrocytes in Response to a Brief Period of Hypoxia and Hypercapnia. Cardiovasc. Res..

[78] Ellsworth M.L., Forrester T., Ellis C.G., Dietrich H.H. (1995). The Erythrocyte as a Regulator of Vascular Tone. Am. J. Physiol. Circ. Physiol..

[79] Locovei S., Bao L., Dahl G. (2006). Pannexin 1 in Erythrocytes: Function without a Gap. Proc. Natl. Acad. Sci. USA.

[80] Ransford G.A., Fregien N., Qiu F., Dahl G., Conner G.E., Salathe M. (2009). Pannexin 1 Contributes to ATP Release in Airway Epithelia. Am. J. Respir. Cell Mol. Biol..

[81] Di Virgilio F., Chiozzi P., Ferrari D., Falzoni S., Sanz J.M., Morelli A., Torboli M., Bolognesi G., Baricordi O.R. (2001). Nucleotide Receptors: An Emerging Family of Regulatory Molecules in Blood Cells. Blood.

[82] Öhman J., Kudira R., Albinsson S., Olde B., Erlinge D. (2012). Ticagrelor Induces Adenosine Triphosphate Release from Human Red Blood Cells. Biochem. Biophys. Res. Commun..

[83] Nylander S., Femia E.A., Scavone M., Berntsson P., Asztély A.-K., Nelander K., Löfgren L., Nilsson R.G., Cattaneo M. (2013). Ticagrelor Inhibits Human Platelet Aggregation via Adenosine in Addition to P2Y 12 Antagonism. J. Thromb. Haemost..

[84] Kirby B.S., Sparks M.A., Lazarowski E.R., Domowicz D.A.L., Zhu H., McMahon T.J. (2021). Integrative Cardiovascular Physiology and Pathophysiology: Pannexin 1 Channels Control the Hemodynamic Response to Hypoxia by Regulating O_2_-Sensitive Extracellular ATP in Blood. Am. J. Physiol.-Heart Circ. Physiol..

[85] von Kügelgen I., Wetter A. (2000). Molecular Pharmacology of P2Y-Receptors. Naunyn. Schmiedebergs. Arch. Pharmacol..

[86] Forsberg E.J., Feuerstein G., Shohami E., Pollard H.B. (1987). Adenosine Triphosphate Stimulates Inositol Phospholipid Metabolism and Prostacyclin Formation in Adrenal Medullary Endothelial Cells by Means of P2-Purinergic Receptors. Proc. Natl. Acad. Sci. USA.

[87] Burnstock G. (2017). Purinergic Signaling in the Cardiovascular System. Circ. Res..

[88] Sridharan M., Adderley S.P., Bowles E.A., Egan T.M., Stephenson A.H., Ellsworth M.L., Sprague R.S. (2010). Pannexin 1 Is the Conduit for Low Oxygen Tension-Induced ATP Release from Human Erythrocytes. Am. J. Physiol. Circ. Physiol..

[89] Sridharan M., Bowles E.A., Richards J.P., Krantic M., Davis K.L., Dietrich K.A., Stephenson A.H., Ellsworth M.L., Sprague R.S. (2012). Prostacyclin Receptor-Mediated ATP Release from Erythrocytes Requires the Voltage-Dependent Anion Channel. Am. J. Physiol.-Heart Circ. Physiol..

[90] Bouyer G., Cueff A., Egée S., Kmiecik J., Maksimova Y., Glogowska E., Gallagher P.G., Thomas S.L.Y. (2011). Erythrocyte Peripheral Type Benzodiazepine Receptor/Voltage-Dependent Anion Channels Are Upregulated by Plasmodium Falciparum. Blood.

[91] Marginedas-Freixa I., Alvarez C.L., Moras M., Leal Denis M.F., Hattab C., Halle F., Bihel F., Mouro-Chanteloup I., Lefevre S.D., Le Van Kim C. (2018). Human Erythrocytes Release ATP by a Novel Pathway Involving VDAC Oligomerization Independent of Pannexin-1. Sci. Rep..

[92] Srihirun S., Sriwantana T., Unchern S., Kittikool D., Noulsri E., Pattanapanyasat K., Fucharoen S., Piknova B., Schechter A.N., Sibmooh N. (2012). Platelet Inhibition by Nitrite Is Dependent on Erythrocytes and Deoxygenation. PLoS ONE.

[93] Leo F., Suvorava T., Heuser S.K., Li J., Lobue A., Barbarino F., Piragine E., Schneckmann R., Hutzler B., Good M.E. (2021). Red Blood Cell and Endothelial ENOS Independently Regulate Circulating Nitric Oxide Metabolites and Blood Pressure. Circulation.

[94] Yang Z., Venardos K., Jones E., Morris B.J., Chin-Dusting J., Kaye D.M. (2007). Identification of a Novel Polymorphism in the 3′UTR of the L-Arginine Transporter Gene SLC7A1. Circulation.

[95] Grupper A., Shashar M., Bahry D., Pri-Paz Y., Tur O.B., Levi S., Chernichovski T., Chernin G., Schwartz I.F. (2013). Cyclosporine Attenuates Arginine Transport, in Human Endothelial Cells, through Modulation of Cationic Amino Acid Transporter-1. Am. J. Nephrol..

[96] Grau M., Pauly S., Ali J., Walpurgis K., Thevis M., Bloch W., Suhr F. (2013). RBC-NOS-Dependent S-Nitrosylation of Cytoskeletal Proteins Improves RBC Deformability. PLoS ONE.

[97] Barbarino F., Wäschenbach L., Cavalho-Lemos V., Dillenberger M., Becker K., Gohlke H., Cortese-Krott M.M. (2021). Targeting Spectrin Redox Switches to Regulate the Mechanoproperties of Red Blood Cells. Biol. Chem..

[98] Porro B., Eligini S., Veglia F., Lualdi A., Squellerio I., Fiorelli S., Giovannardi M., Chiorino E., Dalla Cia A., Crisci M. (2014). Nitric Oxide Synthetic Pathway in Patients with Microvascular Angina and Its Relations with Oxidative Stress. Oxid. Med. Cell. Longev..

[99] Holowatz L.A., Kenney W.L. (2007). Up-Regulation of Arginase Activity Contributes to Attenuated Reflex Cutaneous Vasodilatation in Hypertensive Humans. J. Physiol..

[100] Pernow J., Jung C. (2013). Arginase as a Potential Target in the Treatment of Cardiovascular Disease: Reversal of Arginine Steal?. Cardiovasc. Res..

[101] Quitter F., Figulla H.R., Ferrari M., Pernow J., Jung C. (2013). Increased Arginase Levels in Heart Failure Represent a Therapeutic Target to Rescue Microvascular Perfusion. Clin. Hemorheol. Microcirc..

[102] Masuda H. (2008). Significance of Nitric Oxide and Its Modulation Mechanisms by Endogenous Nitric Oxide Synthase Inhibitors and Arginase in the Micturition Disorders and Erectile Dysfunction. Int. J. Urol..

[103] Hein T.W., Zhang C., Wang W., Chang C.-I., Thengchaisri N., Kuo L. (2003). Ischemia-reperfusion Selectively Impairs Nitric Oxide-Mediated Dilation in Coronary Arterioles: Counteracting Role of Arginase. FASEB J..

[104] Jung C., Gonon A.T., Sjoquist P.-O., Lundberg J.O., Pernow J. (2010). Arginase Inhibition Mediates Cardioprotection during Ischaemia-Reperfusion. Cardiovasc. Res..

[105] Gonon A.T., Jung C., Katz A., Westerblad H., Shemyakin A., Sjöquist P.-O., Lundberg J.O., Pernow J. (2012). Local Arginase Inhibition during Early Reperfusion Mediates Cardioprotection via Increased Nitric Oxide Production. PLoS ONE.

[106] Kövamees O., Shemyakin A., Checa A., Wheelock C.E., Lundberg J.O., Östenson C.-G., Pernow J. (2016). Arginase Inhibition Improves Microvascular Endothelial Function in Patients with Type 2 Diabetes Mellitus. J. Clin. Endocrinol. Metab..

[107] Kövamees O., Shemyakin A., Pernow J. (2014). Effect of Arginase Inhibition on Ischemia-Reperfusion Injury in Patients with Coronary Artery Disease with and without Diabetes Mellitus. PLoS ONE.

